# Mitochondria at the Crossroads of Cardiovascular Disease: Mechanistic Drivers and Emerging Therapeutic Strategies

**DOI:** 10.3390/cells15040372

**Published:** 2026-02-20

**Authors:** Sonila Alia, Gaia Pedriali, Paolo Compagnucci, Yari Valeri, Valentina Membrino, Tiziana Di Crescenzo, Elena Tremoli, Laura Mazzanti, Arianna Vignini, Paolo Pinton, Michela Casella

**Affiliations:** 1 Department of Clinical Sciences, Marche Polytechnic University, 60126 Ancona, Italy; s.alia@staff.univpm.it (S.A.); v.membrino@pm.univpm.it (V.M.); t.dicrescenzo@pm.univpm.it (T.D.C.); m.casella@staff.univpm.it (M.C.); 2 GVM Care & Research, Maria Cecilia Hospital, 48033 Cotignola, Italy; gpedriali@gvmnet.it (G.P.); etremoli@gvmnet.it (E.T.); paolo.pinton@unife.it (P.P.); 3 Department of Cardiology and Arrhythmology Clinic, Marche University Hospital, 60126 Ancona, Italy; paolocompagnucci1@gmail.com; 4 Department of Biomedical Sciences and Public Health, Marche Polytechnic University, 60126 Ancona, Italy; yarivaleri1@gmail.com; 5 Fondazione Salesi, Ospedale G. Salesi, 60123 Ancona, Italy; 6 Department of Medical Sciences, Section of Experimental Medicine, University of Ferrara, 44121 Ferrara, Italy

**Keywords:** mitochondrial dysfunction, cardiovascular disease, mitochondrial quality control, mitophagy, oxidative stress, mitochondrial signaling, inflammation

## Abstract

Mitochondria are central regulators of cardiac homeostasis, integrating energy production, redox balance, calcium handling, and innate immune signaling. In cardiovascular disease (CVD), mitochondrial dysfunction acts as a unifying mechanism connecting oxidative stress, metabolic inflexibility, inflammation, and structural remodeling. Disturbances in mitochondrial quality control—encompassing fusion–fission dynamics, PINK1/Parkin- and receptor-mediated mitophagy, biogenesis, and proteostasis—compromise mitochondrial integrity and amplify cardiomyocyte injury. Excess reactive oxygen species, mitochondrial DNA release, and calcium overload further activate cGAS–STING, NLRP3 inflammasomes, and mPTP-driven cell death pathways, perpetuating maladaptive remodeling. Therapeutic strategies targeting mitochondrial dysfunction have rapidly expanded, ranging from mitochondria-targeted antioxidants (such as MitoQ and SS-31), nutraceuticals, metabolic modulators (SGLT2 inhibitors, metformin), and mitophagy or biogenesis activators to innovative approaches including mtDNA editing, nanocarrier-based delivery, and mitochondrial transplantation. These interventions aim to restore organelle structure, improve bioenergetics, and reestablish balanced quality control networks. This review integrates recent mechanistic insights with emerging translational evidence, outlining how mitochondria function as bioenergetic and inflammatory hubs in CVD. By synthesizing established and next-generation therapeutic strategies, it highlights the potential of precision mitochondrial medicine to reshape the future management of cardiovascular disease.

## 1. Introduction

Cardiovascular diseases (CVDs), a group of disorders of the heart and blood vessels encompassing conditions like coronary heart disease, stroke, and heart failure, remain the leading cause of mortality worldwide, accounting for more than 19.8 million deaths in 2022 (WHO, 2025) [1]. On an annual basis, the American Heart Association (AHA), in collaboration with the National Institutes of Health (NIH), releases updated statistics on cardiovascular and cerebrovascular diseases. These reports highlight the prevalence and trends of major risk determinants, ranging from behavioral aspects such as smoking, diet, physical activity, sleep, and obesity, to clinical factors including blood pressure, cholesterol, glucose regulation, and metabolic syndrome (AHA, 2025) [2]. Lifestyle-related risk factors remain highly prevalent: global obesity has tripled in the last four decades, affecting more than 650 million adults, while nearly 25% of adults do not meet recommended activity levels. These conditions not only elevate cardiovascular risk but also induce mitochondrial dysfunction, including impaired oxidative metabolism, increased ROS generation, and defective mitophagy, thereby reinforcing the mechanistic framework discussed in this review. Beyond their clinical consequences, CVDs impose enormous economic and societal costs, representing one of the largest healthcare expenditures globally [3,4,5,6].

Mitochondria are vital to cardiac physiology, as they are responsible for generating over 90% of the ATP required for cardiac contraction through oxidative phosphorylation [7]. The myocardium requires a continuous supply of energy while simultaneously maintaining the capacity to adapt to fluctuations in workload and substrate availability. These organelles facilitate this adaptation by efficiently converting diverse metabolic substrates into ATP. Under physiological conditions, fatty acids represent the predominant energy source, providing approximately 60–90% of myocardial ATP production [8]. Following uptake from the circulation, fatty acids are imported into mitochondria and oxidized through β-oxidation, yielding acetyl-CoA. This metabolite subsequently enters the tricarboxylic acid (TCA) cycle, generating reducing equivalents (NADH and FADH_2_) that transfer electrons to the electron transport chain (ETC), thereby sustaining oxidative phosphorylation and ATP synthesis.

Glucose metabolism proceeds via cytosolic glycolysis, producing pyruvate, which is subsequently converted into acetyl-CoA by the pyruvate dehydrogenase complex. This pathway also contributes to the TCA cycle and ETC activity. The utilization of fatty acids and glucose is tightly coordinated through the Randle cycle, a reciprocal regulatory mechanism that ensures efficient allocation of available energetic substrates [9].

In addition to fatty acids and glucose, alternative fuels such as ketone bodies, amino acids, and lactate can also be oxidized to support ATP generation. Although their contribution under basal conditions is relatively modest, these substrates assume greater significance in response to metabolic stress or pathological states [8].

In addition to energy production, mitochondria regulate calcium homeostasis, apoptosis, and redox balance. Dysfunctional mitochondria contribute to the excess production of mitochondrial reactive oxygen species (mROS), mitochondrial DNA (mtDNA) mutations, metabolic derangements, and activation of cell death pathways. Collectively, these processes drive the pathogenesis and progression of CVD [6]. Although the discussion above focuses on cardiac mitochondria, mitochondrial dysfunction similarly affects vascular tissues. Oxidized LDL activates endothelial inflammatory pathways and induces mitochondrial stress, demonstrating that mitochondrial dysfunction contributes not only to myocardial injury but also to systemic vascular pathology [10,11].

Although systemic antioxidant therapies have been extensively studied, their limited efficacy in clinical trials underscores the need for mitochondria-specific approaches. Mitochondria-targeted antioxidants (MTAs) and emerging mitochondrial therapies represent a promising paradigm for combating oxidative stress and restoring mitochondrial function [12].

This review aims to integrate mechanistic insights into mitochondrial dysfunction with emerging therapeutic strategies, highlighting mitochondria as bioenergetic, signaling, and inflammatory hubs in cardiovascular disease and as targets for precision medicine approaches.

Although mitochondrial dysfunction has been extensively studied in cardiovascular disease, many investigations have focused on isolated pathways. However, mitochondria function as integrated bioenergetic and signaling hubs where energy metabolism, redox signaling, calcium homeostasis, immune activation, and quality control are tightly interconnected. As a result, therapeutic strategies targeting single mitochondrial processes have often shown limited clinical efficacy. Integrating mechanistic insights with emerging therapeutic approaches is therefore essential to address mitochondrial dysfunction at a systems level and restore mitochondrial homeostasis.

Accordingly, this review is organized to first present the mechanisms regulating mitochondrial dynamics and quality control, then examine how their dysregulation contributes to cardiovascular pathophysiology, and finally discuss current and emerging therapeutic strategies targeting mitochondrial dysfunction.

## 2. Mitochondrial Dynamics and Quality Control

Mitochondrial quality control (MQC) represents a highly coordinated system of mechanisms that preserve organelle integrity, sustain metabolic flexibility, and regulate cell fate under both physiological and pathological conditions. Core processes include mitochondrial fusion and fission, mitophagy, biogenesis, and proteostasis. Beyond these classical mechanisms, recent advances have highlighted additional layers of regulation involving mitochondria–endoplasmic reticulum (ER) contacts, mtDNA signaling, and innate immune pathways, underscoring MQC as a central hub in cardiovascular homeostasis [13,14,15].

Accordingly, this section focuses on mitochondrial dynamics, mitophagy, biogenesis, and associated signaling pathways, which collectively regulate mitochondrial homeostasis and contribute to cellular resilience or vulnerability in cardiovascular disease.

### 2.1. Fusion and Fission Allow for the Exchange of Mitochondrial Contents and Protect Against Localized Damage

Mitochondrial fusion and fission are dynamic processes that maintain the structural and functional integrity of the mitochondrial network. Fusion enables the mixing of mitochondrial membranes and matrix contents, thereby compensating for local defects in mtDNA or proteins and enhancing overall bioenergetic efficiency. This process is orchestrated by the GTPases mitofusin-1 (MFN1) and mitofusin-2 (MFN2), which tether adjacent outer membranes, and optic atrophy protein 1 (OPA1), which regulates inner membrane fusion and cristae architecture [16,17]. Fusion not only promotes metabolic flexibility but also supports resistance to stress by maintaining a connected network capable of distributing metabolites and signaling molecules [18].

In contrast, fission allows for the division of mitochondria into smaller units, a process essential for organelle distribution during cell division and for the selective removal of dysfunctional compartments. The primary effector of fission is dynamin-related protein 1 (DRP1), which is recruited from the cytosol to the outer mitochondrial membrane (OMM) through adaptors including FIS1, mitochondrial fission factor (MFF), and MiD49/51. Mitochondrial dynamics occur preferentially at sites of close apposition between the ER and mitochondria, known as mitochondria-associated membranes (MAMs). These structures function as spatial platforms that coordinate the recruitment of the fission and fusion machinery, including DRP1, and are therefore essential for the initiation of mitochondrial scission [16,19]. Beyond their role in mitochondrial dynamics, MAMs serve as multifunctional regulatory hubs involved in Ca^2+^ transfer and homeostasis, phospholipid and cholesterol metabolism, mitophagy initiation, ROS signaling, bioenergetic control, and apoptotic pathways [20,21,22]. Fission plays a crucial quality control role by segregating damaged portions of the network that can then be targeted for mitophagy [17,23].

**Figure 1:**
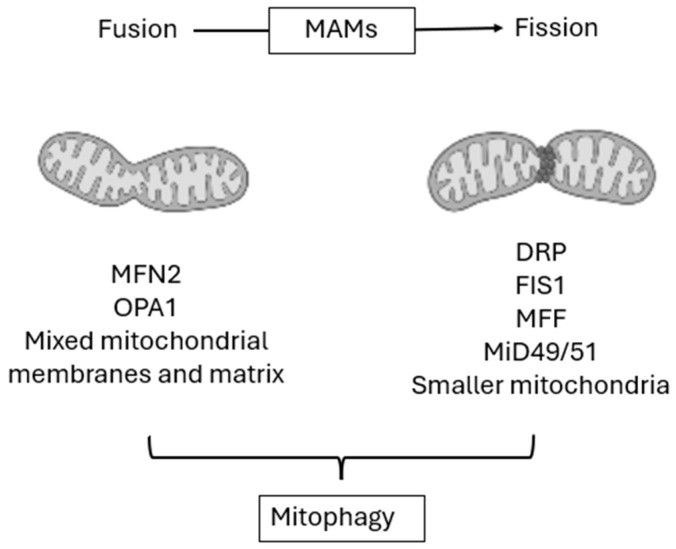
Mitochondrial fusion and fission in quality control. Fusion, mediated by MFN2 and OPA1, promotes the mixing of mitochondrial membranes and matrix contents, whereas fission, driven by DRP1 and its adaptors, generates smaller mitochondrial units. These processes are coordinated at MAMs and contribute to mitochondrial quality control and mitophagy.

However, dysregulated fission is detrimental: excessive fragmentation results in impaired oxidative phosphorylation, overproduction of ROS, and initiation of apoptotic pathways, phenomena that are strongly implicated in ischemia–reperfusion injury, cardiomyocyte death, and progression of heart failure [13,14,24]. Recent studies have further highlighted the role of stress-responsive isoforms of OPA1, processed by the proteases OMA1 and YME1L, in modulating cristae remodeling and shifting the balance between fusion and fission. Under stress, OPA1 cleavage can destabilize cristae structure, facilitating cytochrome c release and apoptosis [13,25]. Disruption of MAMs integrity has been linked to altered calcium homeostasis, mitochondrial fragmentation, and impaired metabolic signaling [14,19]. Together, these observations underscore that mitochondrial dynamics are not isolated processes, but are integrated with inter-organelle communication, metabolic adaptation, and cell survival mechanisms. These coordinated fusion–fission events, occurring preferentially at MAMs, are schematically summarized in Figure 1.

### 2.2. Mitophagy Selectively Removes Damaged Mitochondria

Mitophagy is a selective form of autophagy that eliminates dysfunctional or superfluous mitochondria, thereby preventing the accumulation of damaged organelles that could otherwise compromise bioenergetics and promote oxidative stress. The canonical PINK1/Parkin pathway represents a key mechanism of mitochondrial quality control, mediating ubiquitin-dependent mitophagy. Although this pathway has been extensively studied in neurodegeneration, increasing evidence supports its important role in the heart, a tissue characterized by high energetic demand and susceptibility to mitochondrial dysfunction [26,27]. Under conditions such as ischemia/reperfusion injury or pressure overload, impaired PINK1/Parkin signaling leads to the accumulation of damaged mitochondria, excessive ROS production, and progression toward heart failure [26,28]. Conversely, activation of PINK1/Parkin-mediated mitophagy promotes cardioprotection by preserving mitochondrial integrity, limiting oxidative stress, and enhancing cellular survival under diverse metabolic challenges, including ischemia–reperfusion injury, hyperglycemia and fatty-acid overload, Ca^2+^ dysregulation, hypoxia, mtDNA damage, redox imbalance, and inflammatory stress [29,30,31,32].

In addition to this ubiquitin-dependent system, receptor-mediated mitophagy pathways have gained prominence. Proteins such as BNIP3, NIX (BNIP3L), and FUNDC1, located on the OMM, directly interact with LC3 through their LC3-interacting region (LIR) motifs. These pathways are particularly important under hypoxia and ischemia, where hypoxia-inducible factor 1α (HIF-1α) upregulates BNIP3/NIX, thereby enhancing mitophagy independently of Parkin [13,23]. FUNDC1-mediated mitophagy is regulated by phosphorylation status, providing a fine-tuned mechanism of adaptation to acute and chronic stress [33].

Beyond these classical mechanisms, mitochondria-derived vesicles (MDVs) represent an additional layer of mitochondrial quality control. MDVs bud off from mitochondria carrying selectively oxidized proteins or lipids and traffic them to lysosomes or peroxisomes for degradation. This process offers a rapid, targeted response to localized oxidative damage, sparing the entire organelle from wholesale elimination. The role of MDVs in cardiovascular disease is increasingly appreciated, as they provide a first-line defense that may reduce reliance on bulk mitophagy during ischemic stress [34,35].

Recent studies have also identified additional regulatory elements that modulate mitophagy. Cardiolipin externalization from the inner mitochondrial membrane (IMM) to the OMM acts as an “eat-me” signal, facilitating recognition by autophagic machinery [36]. Moreover, MAMs have been shown to facilitate mitophagy initiation, highlighting the importance of inter-organelle communication in quality control [37].

Dysregulated mitophagy contributes to pathology as insufficient mitophagy results in accumulation of defective mitochondria, elevated ROS, and cell death, whereas excessive mitophagy may deplete mitochondrial content, impairing energy production. Thus, maintaining a balanced level of mitophagy is critical for cardiac homeostasis, as exemplified by studies showing that impairment of mitophagy/autophagy accompanies calcific aortic valve stenosis and favors cell death and disease severity [38]. Therapeutically, enhancing mitophagy has shown protective effects in models of ischemia–reperfusion and heart failure, and pharmacological modulators (e.g., urolithin A, spermidine) are being explored as candidate interventions to restore mitochondrial quality control [39,40].

### 2.3. Mitochondrial Biogenesis Counterbalances Mitophagy by Generating New Organelles

Mitochondrial biogenesis is a fundamental adaptive process that counterbalances mitophagy by generating new organelles, thereby sustaining mitochondrial mass and functional capacity. This process is orchestrated by the transcriptional coactivator peroxisome proliferator–activated receptor γ coactivator-1α (PGC-1α), which integrates environmental and metabolic cues to drive the expression of nuclear-encoded mitochondrial genes. Downstream, nuclear respiratory factors (NRF1/2) and mitochondrial transcription factor A (TFAM) coordinate transcription and replication of mtDNA, ensuring proper organelle assembly [13,41].

Mitochondrial biogenesis is tightly regulated by cellular energy and redox signaling pathways. The energy sensor AMP-activated protein kinase (AMPK) and the NAD+-dependent sirtuins (SIRT1, SIRT3) promote PGC-1α activation through phosphorylation and deacetylation, respectively [42]. Physiological stimuli such as endurance exercise, caloric restriction, and thermal stress enhance biogenesis, while chronic metabolic disorders (e.g., obesity, diabetes) suppress it [43]. Importantly, reduced mitochondrial biogenesis has been implicated in heart failure, where impaired PGC-1α/NRF/TFAM signaling contributes to diminished oxidative capacity and energetic failure in cardiomyocytes [44].

Parallel to organelle renewal, mitochondrial proteostasis mechanisms safeguard protein quality within the matrix and membranes. Chaperones such as HSP60 and mtHSP70 assist protein folding and prevent aggregation, while proteases including LONP1, ClpXP, and AAA proteases (YME1L, AFG3L2) degrade oxidized or misfolded proteins [45]. The mitochondrial unfolded protein response (mtUPR) serves as an adaptive transcriptional program to restore proteostasis, upregulating chaperones and proteases in response to protein misfolding stress [14,46]. Beyond classical PGC-1α–driven regulation, emerging evidence shows that mitochondrial biogenesis is controlled by multiple interconnected layers. First, mitochondrial–nuclear communication through retrograde signaling—mediated by ROS, the NAD^+^/NADH ratio and metabolic intermediates—modulates nuclear transcriptional programs according to mitochondrial status [47]. In parallel, structural regulators such as TFAM contribute not only to mtDNA transcription but also to nucleoid stabilization, thereby safeguarding mitochondrial genome integrity [47]. Epigenetic processes, including histone acetylation and methylation as well as non-coding RNAs such as miR-378 and miR-696, further fine-tune PGC-1α activity and endocrine–metabolic adaptation [48]. Finally, sex-specific regulation has also been described: estrogen receptor signaling enhances PGC-1α activation and may promote cardioprotection by stimulating mitochondrial renewal [49]. Therapeutically, several approaches aim to stimulate mitochondrial biogenesis and proteostasis. Pharmacological agents such as metformin and NAD^+^ precursors, together with nutraceutical polyphenols including resveratrol, converge on AMPK–SIRT–PGC-1α signaling to improve mitochondrial function in preclinical models of cardiovascular disease [50,51]. Although metformin is frequently discussed for its beneficial effects on cardiac metabolism, accumulating evidence indicates that it exerts a direct inhibitory action on mitochondrial respiration. By limiting ETC activity at the level of complex I, metformin reduces oxidative ATP production and promotes a compensatory shift toward glycolytic metabolism, with a concomitant increase in lactate generation. Importantly, recent studies suggest that these effects follow a biphasic and context-dependent pattern, conferring protection under ischemic or metabolic stress conditions while potentially impairing mitochondrial efficiency when sustained or excessive [52,53].

Additionally, boosting the mtUPR has been proposed as a strategy to enhance organelle resilience under stress. For example, Cilleros-Holgado et al. (2023) [54] explored the modulation of the mtUPR as a potential therapeutic target in mitochondrial diseases, although clinical translation remains in its early stages.

### 2.4. Beyond Energy Production, Mitochondria Are Increasingly Recognized as Signaling Organelles

Beyond their classical role in ATP synthesis, mitochondria function as versatile signaling platforms that integrate stress responses, immune activation, and cell fate decisions. One prominent mechanism involves the release of mtDNA into the cytosol, which is sensed by cyclic GMP–AMP synthase (cGAS) and subsequently activates the STING pathway. This cascade triggers type I interferon and pro-inflammatory cytokine production, thereby linking MQC failure to innate immune activation and chronic [35,55]. In cardiovascular disease, persistent mtDNA release contributes to sterile inflammation, adverse remodeling, and progression toward heart failure [56].

In addition to mtDNA, mitochondria release other damage-associated molecular patterns (DAMPs), including cardiolipin, TFAM, mtROS, cytochrome c and N-formyl peptides, all of which can trigger Toll-like receptor (TLR) or activate the inflammasome [57]. In particular, the NLRP3 inflammasome is activated by mitochondrial danger signals such as mtROS and mtDNA, leading to caspase-1 activation and IL-1β maturation—processes implicated in atherosclerosis, myocardial infarction and heart failure [58,59]. More recent evidence indicates that this activation is tightly regulated at MAMs, where ER–mitochondria crosstalk, Ca^2+^ transfer and local ROS production facilitate NLRP3 assembly [20,60].

Mitochondria also act as redox signaling hubs. ROS generated at complexes I and III of the ETC function as second messengers in physiological adaptation, but under stress conditions excessive ROS propagate oxidative damage and activate maladaptive signaling cascades such as NF-κB and MAPKs (23). Moreover, TCA cycle metabolites (e.g., succinate, fumarate, α-ketoglutarate) influence nuclear gene expression by modulating epigenetic enzymes such as histone and DNA demethylases, thereby integrating mitochondrial metabolism with nuclear transcriptional programs [61].

A central regulator of mitochondrial signaling is the mitochondrial permeability transition pore (mPTP). Under conditions of severe stress (e.g., Ca^2+^ overload, oxidative injury), sustained mPTP opening results in loss of membrane potential, swelling, and release of pro-apoptotic factors such as cytochrome c and apoptosis-inducing factor (AIF), leading to apoptosis or necrosis [62,63]. Importantly, transient mPTP flickering may also serve physiological signaling roles, highlighting its dual nature [64,65]. In ischemia–reperfusion and heart failure, persistent mPTP activation exacerbates cardiomyocyte death and adverse remodeling [66,67].

Collectively, these findings underscore mitochondria as active signaling organelles that govern the balance between cellular adaptation and pathology. Aberrant mitochondrial signaling contributes to chronic inflammation, innate immune activation, and cell death pathways in cardiovascular disease. In particular, mitochondrial stress can promote the release of mitochondrial-damage-associated molecular patterns, such as mtDNA, which activate innate immune sensors including the cGAS–STING pathway and amplify inflammatory signaling in cardiac and vascular cells [68,69]. In parallel, mitochondrial dysfunction and excessive mitochondrial ROS production have been linked to inflammasome activation, most notably NLRP3, thereby sustaining sterile inflammation and adverse cardiac remodeling [70,71]. Accordingly, therapeutic strategies aimed at modulating cGAS–STING signaling, inflammasome activation, and mPTP opening are under active investigation as potential interventions for ischemic and inflammatory cardiac disorders [65,72,73].

### 2.5. Targeting Mitochondrial Quality Control as Therapy

Although multiple strategies targeting MQC have shown cardioprotective effects, the strength of supporting evidence varies considerably among approaches. Most interventions described herein are supported predominantly by experimental and preclinical studies, while clinical translation remains limited or inconclusive in many cases, underscoring the need for a critical and comparative evaluation of their therapeutic potential [13,14,24].

Targeting MQC has emerged as a promising therapeutic avenue for cardiovascular diseases, as interventions that restore mitochondrial balance can attenuate energetic failure, reduce oxidative damage, and prevent maladaptive remodeling. Strategies under investigation include modulation of mitochondrial dynamics, mitophagy, biogenesis, and lipid remodeling, all of which converge to maintain organelle integrity and function.

Pharmacological inhibition of DRP1-mediated fission has demonstrated cardioprotective effects in preclinical models. The small molecule Mdivi-1 prevents excessive fragmentation, reduces ROS, and improves cell survival following ischemia–reperfusion [24]. More selective and clinically viable DRP1 inhibitors are being developed, although their safety and specificity remain to be fully validated [74]. Despite robust cardioprotective effects observed in experimental models, pharmacological modulation of mitochondrial fission remains challenged by issues of target specificity, off-target effects, and the lack of consistent clinical validation [24,72].

Enhancing mitophagy represents another therapeutic approach. Compounds such as urolithin A, spermidine, and agents targeting the PINK1/Parkin axis promote the clearance of damaged mitochondria, thereby improving mitochondrial turnover and cardiac function in models of heart failure and aging [13,32,75]. Compared with interventions targeting mitochondrial dynamics, strategies aimed at enhancing mitophagy or mitochondrial biogenesis may offer greater adaptability to metabolic stress; however, current evidence supporting these approaches remains largely preclinical, and their long-term efficacy in cardiovascular patients has yet to be established [13,32,44].

Similarly, BNIP3/FUNDC1-mediated pathways are being explored for their ability to protect cardiomyocytes under hypoxia [76,77]. Stimulation of mitochondrial biogenesis through activation of the AMPK–SIRT–PGC-1α axis has been proposed to restore energetic capacity. Interventions such as resveratrol, NAD+ precursors (nicotinamide riboside, NMN), and metformin activate this pathway and improve mitochondrial density and efficiency in both preclinical and early clinical studies [50,51].

Cardiolipin stabilization is a particularly attractive therapeutic target. Cardiolipin is essential for maintaining cristae architecture and for initiating mitophagy, as its externalization on the OMM recruits LC3 [36]. During ischemic stress, cardiolipin oxidation destabilizes respiratory supercomplexes, facilitates mPTP opening, and amplifies cell-death pathways [78,79]. Compounds such as melatonin can attenuate cardiolipin peroxidation and preserve mitochondrial structure and function [80]. The tetrapeptide elamipretide (SS-31) binds cardiolipin, preserving cristae integrity and improving mitochondrial respiration. Early-phase clinical trials in patients with heart failure and ischemic cardiomyopathy have shown improved left ventricular function and exercise capacity, although larger trials are required to confirm efficacy [81,82]. Other emerging avenues include modulation of the mtUPR to enhance proteostasis [54], targeting NLRP3 inflammasome activation with inhibitors such as MCC950 [83], and pharmacological modulation of the cGAS–STING pathway, which is currently under preclinical evaluation in cardiac inflammation models [84].

Collectively, these findings suggest that fine-tuning MQC—rather than fully blocking or stimulating single pathways—may hold the most significant therapeutic promise. Approaches that dynamically balance fission/fusion, mitophagy/biogenesis, and lipid/proteostasis could pave the way for precision mitochondrial medicine in cardiovascular disease.

Pharmacological inhibitors of DRP1-mediated fission, activators of mitophagy, stimulators of mitochondrial biogenesis, and agents that prevent mPTP opening are being investigated as strategies to restore mitochondrial balance in heart failure and ischemic injury [14,84]. Moreover, stabilization of cardiolipin has been proposed as a novel intervention point [36,78,79]. Although inhibition of mPTP opening has shown strong cardioprotective effects in experimental models, clinical translation has been limited. This discrepancy likely reflects the complexity of ischemia–reperfusion injury in humans, where multiple parallel injury pathways and patient-related factors reduce the effectiveness of targeting a single mitochondrial mechanism [14,24].

Among the pharmacological approaches targeting the mPTP, cyclosporin A has been extensively evaluated in clinical settings. Despite the strong cardioprotective effects observed in experimental models, pharmacological inhibition of the mitochondrial permeability transition pore has yielded limited benefits in clinical settings. In the case of cyclosporin A, the lack of efficacy in patients likely reflects the complexity of ischemia–reperfusion injury in humans, where the timing of intervention, drug bioavailability within the myocardium, and the presence of comorbidities and concomitant therapies critically influence therapeutic outcomes. Moreover, targeting mPTP alone may be insufficient to counteract the multiple parallel injury pathways activated during acute cardiac ischemic events, underscoring the challenges of translating mitochondrial cardioprotection into clinical practice [85]. Although multiple strategies targeting mitochondrial quality control have shown cardioprotective effects, the strength of supporting evidence varies considerably among approaches. Most interventions described herein are supported predominantly by experimental and preclinical studies, while clinical translation remains limited or inconclusive in many cases, underscoring the need for a critical and comparative evaluation of their therapeutic potential [13,14,24].

## 3. Mitochondrial Dysfunction in Cardiovascular Pathophysiology

Mitochondrial dysfunction is increasingly recognized as a central driver of cardiovascular pathophysiology, orchestrating oxidative stress, inflammation, calcium imbalance, and metabolic remodeling. These alterations not only impair cardiac energetics, but also activate maladaptive signaling cascades that contribute to arrhythmias, cardiomyopathy, and heart failure [86,87,88,89]. Mitochondrial dysfunction is a key contributor to the development and progression of cardiovascular disease, linking alterations in energy metabolism to oxidative stress, inflammatory responses, and impaired cellular homeostasis [8]. The following subsections outline the principal mechanisms through which mitochondrial dysfunction contributes to cardiovascular pathophysiology.

### 3.1. Oxidative Stress

Oxidative stress is among the earliest and most pernicious indicators of mitochondrial damage. Defects or inefficiencies in the ETC—especially at Complex I and Complex III—cause electrons to “leak” and prematurely reduce molecular oxygen, producing ROS such as superoxide (O_2_^•−^), hydrogen peroxide (H_2_O_2_), hydroxyl radicals (^•^OH), and peroxynitrite (ONOO^−^). These ROS species cause oxidative damage on mitochondrial lipids (lipid peroxidation), proteins (carbonylation, nitration), and mtDNA lesions, undermining membrane integrity, ETC function, and ATP synthesis. Over time, this cascade can trigger intrinsic apoptotic pathways via cytochrome c release and caspase activation [90].

In the setting of myocardial ischemia–reperfusion (I/R), the abrupt reintroduction of oxygen at reperfusion supercharges ROS generation. Multiple mechanisms contribute to this oxidative burst, including reverse electron transport at Complex I fueled by accumulated succinate during ischemia, as well as impaired antioxidant buffering. The resulting high ROS flux exacerbates mPTP opening, mitochondrial swelling, and cardiomyocyte death [91,92,93].

Expanding upon these classical mechanisms, recent studies have elucidated additional layers of ROS-mediated mitochondrial injury and adaptive signaling. During ischemia, the accumulation of succinate represents a critical metabolic event that profoundly influences mitochondrial redox dynamics. Upon reperfusion, the rapid oxidation of succinate at Complex II drives reverse electron transport through Complex I, resulting in a surge of O_2_•^−^ production, a process identified as a key contributor to I/R injury [91,92]. This reverse electron flux establishes a self-reinforcing cycle in which excessive ROS generation further destabilizes electron transport, amplifying oxidative damage and energetic collapse.

Concurrently, the intrinsic antioxidant defenses of mitochondria, including glutathione peroxidases, peroxiredoxins, and the thioredoxin system, become progressively overwhelmed, tipping the redox balance toward a persistent pro-oxidant state. This redox disequilibrium not only enhances mitochondrial lipid and protein oxidation but also interferes with key signaling pathways that regulate metabolic homeostasis [94].

Interestingly, while excessive ROS are deleterious, low to moderate levels can serve as physiological signaling mediators. Under chronic sublethal stress, mROS activate redox-sensitive transcription factors such as NRF2, which upregulate antioxidant gene expression and reinforce stress resistance. This hormetic response, often referred to as “mitohormesis,” reflects the dual nature of ROS as both damaging agents and essential modulators of cellular adaptation [95].

Persistent oxidative stress exerts profound effects on mitochondrial homeostasis, extending far beyond transient redox imbalance. When sustained over time, excessive ROS production disrupts the tightly regulated mechanisms of mitochondrial turnover, particularly biogenesis and mitophagy, thereby impairing the cell’s ability to renew its organelle population. Impaired mitophagy leads to the accumulation of dysfunctional mitochondria, which in turn become chronic sources of ROS, establishing a self-perpetuating feedback loop that amplifies oxidative damage and metabolic inefficiency [31,96].

This maladaptive cycle has been directly implicated in the progression of cardiovascular diseases, including heart failure, I/R injury, and atherosclerosis. In these conditions, mitochondria lose their capacity for efficient oxidative phosphorylation, leading to energetic failure, enhanced apoptotic signaling, and adverse structural remodeling of the myocardium [97]. Furthermore, sustained oxidative damage suppresses mitochondrial biogenesis through inhibition of the PGC-1α/NRF1/2 axis, limiting the transcriptional renewal of mitochondrial genes and exacerbating energy deficits [98].

Recent evidence indicates that oxidative stress is not merely a consequence of mitochondrial dysfunction but acts as a central mechanistic driver of cardiac pathology, especially in the context of I/R injury. This vicious cycle of redox imbalance and structural injury establishes oxidative stress as both a cause and a consequence of mitochondrial decline, highlighting its pivotal role in the pathogenesis of heart failure and other cardiovascular disorders [99].

**Figure 2:**
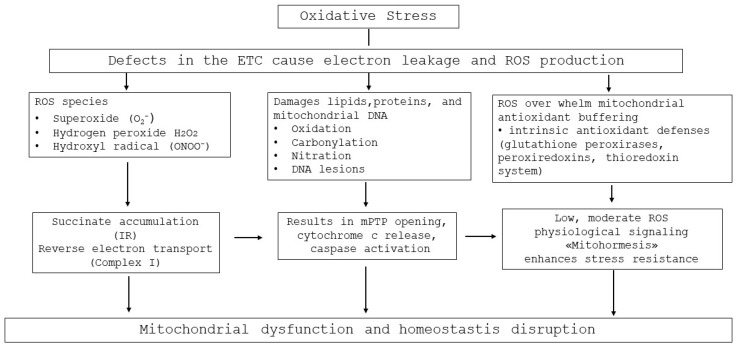
Mitochondrial oxidative stress in cardiovascular disease. Defects in the electron transport chain lead to excessive mitochondrial ROS production, causing oxidative damage to lipids, proteins, and mtDNA. Persistent oxidative stress promotes mPTP opening, impairs mitochondrial turnover, and contributes to energetic failure and cardiac remodeling.

Collectively, these insights underscore that maintaining mitochondrial redox equilibrium and turnover integrity is critical to preventing cardiac dysfunction. Understanding the intertwined pathways of oxidative injury, defective mitophagy, and impaired biogenesis may thus provide the conceptual foundation for developing targeted mitochondria-protective therapies aimed at preserving energetic efficiency and cellular viability in cardiovascular disease. A schematic overview of mitochondrial ROS production, redox imbalance, and downstream consequences in cardiovascular disease is provided in Figure 2.

### 3.2. Inflammation

Inflammation and mitochondrial dysfunction are deeply interwoven in cardiovascular pathophysiology. Mitochondria-derived ROS act as potent upstream activators of NF-κB and MAPK signaling cascades, thereby promoting expression of pro-inflammatory cytokines and adhesion molecules. For example, in vascular cells, NOX4-derived ROS have been shown to activate NF-κB through p38 MAPK signaling, which in turn facilitates NLRP3 inflammasome activation and IL-1β release [100].

Concurrently, when mitochondrial damage is severe, fragments of mtDNA can escape into the cytosol, where it serves as a DAMP. Cytosolic mtDNA is recognized by sensors such as cGAS, which triggers the cGAS–STING pathway and downstream activation of IRF3 and NF-κB, culminating in the production of type I interferons and pro-inflammatory cytokines [84]. This axis provides a mechanistic bridge between mitochondrial stress and chronic sterile inflammation in atherosclerosis, heart failure, and I/R injury.

Support for the role of mtDNA release in driving cGAS-STING-mediated inflammation comes also from studies showing that stressed mitochondria release mtDNA fragments via mPTP or OMM permeabilization. These events facilitate the cytosolic accumulation of mtDNA, which then engages cGAS to initiate inflammatory signaling [101]. Moreover, recent studies have confirmed that mtDNA released during aging or pathological stress can activate cGAS-STING signaling in multiple tissues, contributing to inflammation and disease progression [102].

Thus, mitochondrial oxidative stress does not merely accompany inflammation, it can initiate and amplify innate immune activation through ROS-mediated signaling and mtDNA sensing. In the cardiovascular context, this interplay may sustain vascular inflammation, adverse cardiac remodeling, and the progression of heart failure. Recognizing these interconnections is crucial to design mitochondria-targeted anti-inflammatory strategies in cardiovascular disease [84,87,89].

### 3.3. Calcium Dysregulation

Calcium dysregulation represents a hallmark of mitochondrial dysfunction, exerting profound consequences on cardiomyocyte viability and contractile performance. Under physiological conditions, mitochondria buffer cytosolic calcium through the mitochondrial calcium uniporter (MCU), which couples Ca^2+^ influx to oxidative phosphorylation, thereby matching ATP production to the energetic demands of contraction. However, during pathological states such as I/R, excessive cytosolic and mitochondrial calcium accumulation leads to overactivation of the MCU, resulting in persistent opening of the mPTP. This event causes loss of mitochondrial membrane potential, osmotic swelling, and the release of pro-apoptotic factors, driving both necrotic and apoptotic cell death [103].

Experimental models have shown that genetic or pharmacological inhibition of MCU markedly reduces mitochondrial calcium overload, delays mPTP opening, and mitigates tissue damage during reperfusion [104]. Dysregulation of the MCU complex has also been implicated in cardiac hypertrophy and heart failure, where aberrant calcium handling contributes to energetic failure and maladaptive remodeling [105].

Recent studies have further emphasized that mitochondrial Ca^2+^ overload and oxidative stress are tightly interconnected. ROS not only promote Ca^2+^ accumulation by sensitizing the MCU but also act synergistically to trigger irreversible mPTP opening and mitochondrial collapse [106]. This vicious cycle underlies cardiomyocyte death, contractile dysfunction, and progression toward heart failure [107].

Moreover, in the setting of myocardial ischemia–reperfusion injury, the mPTP serves as a critical integrator of multiple stress signals—oxidative damage, calcium overload, and pH normalization—that converge to determine cell fate. Persistent pore opening accelerates mitochondrial energy collapse and triggers the release of cytochrome c, linking metabolic failure to inflammation and fibrosis [108].

Taken together, these results emphasize that calcium dysregulation and uncontrolled mPTP opening are central mediators of mitochondrial injury in the heart. In particular, the mPTP has been identified as a pivotal node in cardiac dysfunction: its pathological opening collapses the mitochondrial membrane potential, uncouples respiration, halts ATP synthesis, and precipitates necrotic or apoptotic cell death [107]. The structural and mechanistic insights into mPTP function further support its role in ischemia–reperfusion injury and cardioprotection strategies [109]. Indeed, in the cardiovascular disease field, dysregulated mitochondrial calcium uptake and mPTP activation are widely recognized as key drivers of energetic failure, oxidative stress, and remodeling, making the MCU–mPTP axis a compelling therapeutic target [108,110].

### 3.4. Metabolic Remodeling and Its Contribution to Cardiac Dysfunction

In the failing heart, substrate metabolism undergoes a pronounced shift from fatty acid oxidation (FAO) toward increased reliance on glucose metabolism. Initially, this reprogramming may be adaptive, since glucose oxidation requires less oxygen per ATP produced, but over time it leads to a lower net ATP yield, reduced metabolic flexibility, and energetic inefficiency. Evidence from Lopaschuk et al. (2021) shows that mitochondrial oxidative capacity falls in heart failure, and compensatory glycolytic flux increases to maintain ATP supply [111]. The concept of metabolic inflexibility in hypertrophied and failing hearts is also articulated in Ritterhoff et al. (2023), which examines maladaptive rewiring of energy pathways under stress [112]. Additionally, Dato et al. (2024) describe how decreased FAO and the loss of substrate versatility exacerbate cardiac dysfunction in failing myocardium [113].

These changes are not purely compensatory; they aggravate disease progression. The reduced reliance on fatty acid substrates compromises mitochondrial β-oxidation enzyme activity and cofactor balance, thereby promoting further mitochondrial stress and ROS generation. Concurrently, the altered substrate flux perturbs the TCA cycle, disrupting the generation and balance of key intermediates. Emerging metabolomic and signaling studies suggest that TCA cycle metabolites, such as succinate, fumarate, and α-ketoglutarate, do more than fuel bioenergetics; they act as signaling molecules that regulate chromatin modifications, DNA methylation, and stress-response gene expression [61]. Thus, disturbed mitochondrial substrate utilization can propagate maladaptive epigenetic and transcriptional programs that further compromise mitochondrial competency and accelerate pathological remodeling.

Recent reviews emphasize that metabolic remodeling in heart failure is a multifaceted phenomenon: it integrates changes in substrate preference, redox state, mitochondrial function, and signal transduction [114]. In support, was describes how flexibility in fuel use is essential for cardiac health, and how its breakdown in disease is linked to mitochondrial dysfunction and progression of heart failure [113].

**Figure 3:**
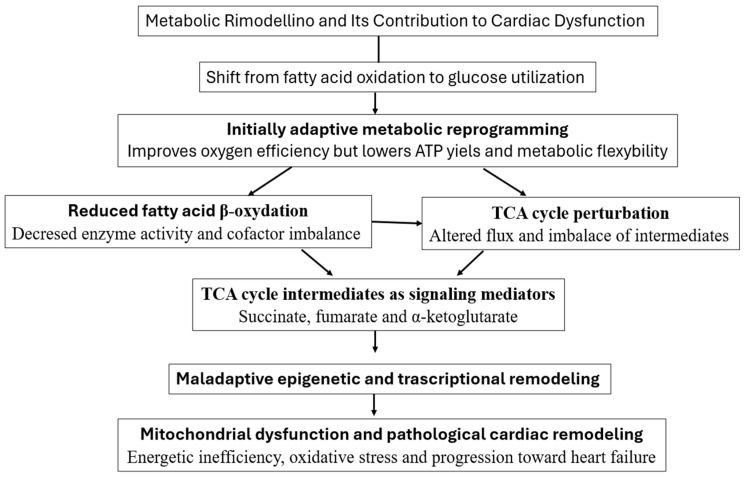
Metabolic remodeling and mitochondrial dysfunction in the failing heart. A shift from fatty acid oxidation toward increased glucose utilization initially improves oxygen efficiency but reduces ATP yield and metabolic flexibility. Reduced β-oxidation and perturbation of the TCA cycle alter the balance of key metabolic intermediates, including succinate, fumarate, and α-ketoglutarate, which act as signaling molecules. These changes promote maladaptive epigenetic and transcriptional remodeling, ultimately contributing to mitochondrial dysfunction, oxidative stress, and pathological cardiac remodeling.

The sequential relationship between altered substrate utilization, TCA cycle perturbation, signaling functions of metabolic intermediates, and maladaptive cardiac remodeling is schematically summarized in Figure 3.

## 4. Therapeutic Strategies

Therapies targeting mitochondrial dysfunction span mitochondria-targeted antioxidants (MTAs), nutraceuticals, pharmacological agents, and emerging biotechnological approaches [89,115,116].

### 4.1. Mitochondria-Targeted Antioxidants

MTAs and related pharmacological approaches constitute a promising strategy to counteract mitochondrial dysfunction. One of the most studied MTAs is MitoQ, a ubiquinone derivative conjugated to triphenylphosphonium (TPP^+^), which accumulates selectively within mitochondria. In preclinical models, chronic MitoQ treatment reverses age-associated arterial stiffness and improves endothelial function by reducing mitochondrial ROS and restoring nitric oxide (NO) bioavailability [117]. In human trials, acute MitoQ intake has been shown to enhance flow-mediated dilation and superoxide dismutase (SOD) activity in patients with peripheral artery disease [118].

Another potent mitochondrial therapy is SS-31 (Elamipretide), a mitochondria-penetrating tetrapeptide that targets cardiolipin in the IMM. SS-31 stabilizes cristae structure, reduces ROS production, and helps preserve ATP synthesis. In animal models, SS-31 mitigates mitochondrial perturbations, enhances respiratory chain efficiency, and corrects supercomplex organization [119]. Moreover, in clinical settings, elamipretide has been tested in Barth syndrome patients; a crossover study indicated improved 6 min walk performance and symptomatic relief [120,121]. In additional trials, short-term elamipretide administration has shown favorable safety and modest improvement in mitochondrial enminergetic capacity relative to placebo [122].

The protein interaction landscape of SS-31 has been mapped: SS-31 interacts with multiple mitochondrial proteins, influencing respiratory efficiency and redox balance [123]. Preclinical studies also show that SS-31 improves mitochondrial morphology, reduces oxidative stress, and enhances function in models of aging and disease [124].

Beyond these well-established compounds, additional mitochondria-targeted antioxidants play equally important roles. MitoTEMPO, a mitochondria-targeted superoxide dismutase mimetic, effectively reduces mitochondrial superoxide levels and is strongly protective in models of vascular dysfunction, atherosclerosis, and hypertension [125,126]. Meanwhile, melatonin, which naturally accumulates in mitochondria, exerts potent antioxidant and anti-inflammatory effects through SIRT3 activation, inhibition of the mPTP, enhancement of ETC efficiency, and suppression of NLRP3 inflammasome activation. Both agents offer mechanistic complementarity to MitoQ and SS-31 by targeting ROS detoxification and inflammatory signaling through distinct pathways [127,128,129].

Recent comprehensive reviews highlight the expanding landscape of mitochondria-directed therapeutic platforms. These encompass emerging TPP^+^-based derivatives, peptide carriers, nanoparticle delivery systems, and next-generation site-specific antioxidants (e.g., SkQ1), many of which show strong potential against oxidative stress–driven cardiovascular pathology. Collectively, these advancements underscore the therapeutic promise of mitochondria-targeted anti-inflammatory agents and support the rationale for expanding pharmacological investigations in CVDs [130].

### 4.2. Lifestyle and Nutraceuticals

Lifestyle interventions and nutraceutical supplementation represent the fundamental non-pharmacological strategies for preserving mitochondrial health and counteracting cardiovascular dysfunction. Among lifestyle factors, regular physical exercise is one of the most potent physiological stimuli for improving mitochondrial performance. Endurance and moderate-intensity aerobic exercise activate energy-sensing pathways such as AMP-activated protein kinase (AMPK), Ca^2+^/calmodulin-dependent kinases (CaMKs), and p38 MAPK, which converge on the transcriptional coactivator PGC-1α. Activated PGC-1α coordinates nuclear and mitochondrial gene expression, promoting mitochondrial biogenesis, increased oxidative capacity, and improved antioxidant defenses [131].

This adaptive remodeling enhances ATP generation and the expression of antioxidant enzymes such as SOD2 and catalase, thereby reinforcing mitochondrial resilience to oxidative stress [132].

Beyond bioenergetic improvement, exercise reduces mROS production by optimizing ETC efficiency and promoting mitophagy via the PINK1/Parkin pathway, which selectively removes damaged organelles and preserving mitochondrial quality [133]. These mechanisms collectively contribute to improved endothelial function, reduced arterial stiffness, and protection against age-related cardiac decline [86,134,135,136]. Dietary patterns exert profound effects on mitochondrial homeostasis, influencing several processes central to cardiovascular health. Nutrient composition and diet quality modulate mitochondrial biogenesis, respiratory chain efficiency, ROS production, and lipid-handling capacity. For instance, Mediterranean-style dietary patterns, rich in unsaturated fats, antioxidants and polyphenols, enhance PGC-1α–mediated mitochondrial biogenesis, improve oxidative phosphorylation, and reduce mitochondrial oxidative stress, thereby supporting endothelial and cardiac metabolic function. In contrast, Western dietary patterns high in saturated fats and refined sugars promote mitochondrial dysfunction, increased ROS generation, impaired OXPHOS, and inflammatory activation, all of which contribute to vascular dysfunction, atherosclerosis, and cardiometabolic disease [137,138].

Sleep quantity and circadian alignment profoundly influence mitochondrial physiology. Insufficient or fragmented sleep reduces mitochondrial respiratory capacity, promotes oxidative stress, impairs NAD^+^ homeostasis, and exacerbates cardiac vulnerability, particularly in cardiometabolic settings [139,140,141]. Disruption of circadian rhythms further destabilizes mitochondrial dynamics by altering clock-controlled pathways that regulate mitobiogenesis, fusion–fission balance, and mitophagy [142,143,144]. Moreover, metabolic–clock interactions involving NAD^+^ balance connect chronic sleep restriction to exaggerated inflammatory signaling and mitochondrial redox dysfunction [145,146].

In parallel, an expanding body of evidence supports the role of nutraceuticals—bioactive compounds derived from food—in modulating mitochondrial function and oxidative homeostasis. Polyphenols such as resveratrol, curcumin, and quercetin are among the most extensively studied. Resveratrol activates SIRT1, leading to deacetylation and activation of PGC-1α, thereby stimulating mitochondrial biogenesis and enhancing oxidative phosphorylation. Moreover, resveratrol suppresses NADPH oxidase activity and inhibits NF-κB signaling, attenuating vascular inflammation and endothelial dysfunction [147,148,149].

Similarly, curcumin exerts multifaceted mitochondrial benefits by improving ETC efficiency, reducing lipid peroxidation, and stimulating NRF2-mediated antioxidant responses. Preclinical studies demonstrate that curcumin supplementation protects against cardiac hypertrophy and I/R injury through attenuation of mitochondrial oxidative damage and modulation of apoptotic signaling [150,151].

Quercetin, another dietary flavonoid, enhances mitochondrial biogenesis and dynamics through AMPK-PGC-1α activation while promoting mitophagy and maintenance of mitochondrial membrane potential [152].

Meanwhile, omega-3 fatty acids such as eicosapentaenoic acid (EPA) and docosahexaenoic acid (DHA) modulate mitochondrial membrane composition, improving fluidity and ETC efficiency while reducing cardiolipin oxidation. Supplementation with omega-3s has been shown to lower systemic inflammation and improve cardiac mitochondrial respiration, particularly in models of metabolic syndrome and heart failure [153,154].

Among endogenous mitochondrial cofactors, coenzyme Q10 (CoQ10) stands out for its dual role as an electron carrier in the ETC and as a lipid-soluble antioxidant. CoQ10 supplementation replenishes mitochondrial ubiquinone pools, decreases lipid peroxidation, and improves ATP synthesis efficiency. Clinical trials have reported that CoQ10 improves endothelial function and left ventricular performance in patients with heart failure and hypertension [155,156,157]. Recent systematic reviews emphasize that nutraceutical strategies combining polyphenols, omega-3s, and CoQ10 may synergistically restore redox balance and improve mitochondrial integrity in cardiovascular disorders [149].

Melatonin represents another mitochondria-active nutraceutical with well-characterized cardiometabolic benefits. Beyond its role as a circadian hormone, melatonin accumulates within mitochondria, where it enhances ETC efficiency, reduces ROS generation, stabilizes mitochondrial membrane potential, and limits mPTP opening. Through SIRT3 activation and NLRP3-inflammasome suppression, melatonin exerts potent antioxidant and anti-inflammatory actions that contribute to myocardial protection and improved endothelial resilience [71,129].

Creatine also contributes to mitochondrial homeostasis by stabilizing ATP buffering and supporting the phosphocreatine shuttle, thereby improving energetic resilience during cardiac stress. Evidence suggests that creatine supplementation may benefit conditions characterized by mitochondrial dysfunction [158].

### 4.3. Pharmacological Agents with Mitochondrial Effects

Beyond lifestyle and nutraceutical interventions, several pharmacological agents traditionally used in cardiovascular medicine exhibit beneficial effects on mitochondrial function, redox balance, and bioenergetic efficiency. Among them, sodium-glucose cotransporter-2 inhibitors (SGLT2i) have emerged as a novel class of cardioprotective agents acting partly through mitochondrial modulation. Experimental studies demonstrate that SGLT2i such as empagliflozin and dapagliflozin enhance mitochondrial oxidative phosphorylation, increase ATP production, and reduce excessive ROS generation independently of glycemic control [159,160].

Mechanistically, SGLT2i improve mitochondrial dynamics by preserving fusion–fission balance and attenuating mitochondrial swelling under oxidative stress [161,162]. In both diabetic and non-diabetic heart failure models, these agents normalize mitochondrial membrane potential and upregulate genes involved in oxidative metabolism [163].

Metformin, a cornerstone in metabolic therapy, also exerts mitochondrial protective effects beyond its glucose-lowering properties. Through the activation of AMPK, metformin enhances fatty acid oxidation, inhibits mitochondrial complex I–driven ROS production, and promotes autophagy and mitochondrial biogenesis [164].

Recent evidence indicates that metformin exerts cardioprotective effects during ischemia–reperfusion injury by preserving mitochondrial integrity and preventing excessive mPTP opening. These effects are largely attributed to complex I inhibition, which limits electron leakage and ROS generation during early reperfusion, thereby attenuating oxidative stress and apoptosis in cardiomyocytes [165].

Moreover, metformin activates the AMPK-dependent pathways that promote autophagy, stabilize mitochondrial membrane potential, and support energy homeostasis under hypoxic conditions [53].

Complementary findings from Bu et al. (2022) further highlight the broad cardiometabolic benefits of metformin, including the reduction in oxidative damage, improvement of endothelial function, and mitigation of mitochondrial dysfunction in both diabetic and non-diabetic models of cardiovascular disease [166]. These findings position metformin as an indirect modulator of mitochondrial quality control through AMPK-dependent signaling.

Furthermore, statins and angiotensin-converting enzyme (ACE) inhibitors, while primarily prescribed for lipid-lowering and hemodynamic effects, display pleiotropic actions involving mitochondrial protection and redox regulation. Statins enhance endothelial nitric oxide synthase (eNOS) expression and suppress NADPH oxidase activity, thereby improving endothelial function and reducing ROS production. However, these benefits may depend on dose and treatment duration, as high-dose statin therapy can impair mitochondrial biogenesis [167].

Enhancing mitochondrial proteostasis is emerging as a complementary strategy to maintain mitochondrial function under stress. Activation of the mtUPR increases chaperone activity (HSP60, mtHSP70) and promotes the degradation of misfolded proteins through LONP1 and ClpP proteases [46,168]. Pharmacological induction of mitochondrial stress—such as tetracycline-derived compounds including doxycycline—can activate mtUPR signaling, increase chaperone expression, and improve mitochondrial proteostasis in cellular models [169]. Although evidence in cardiac models remains limited, mtUPR modulation represents a mechanistically complementary approach to existing mitochondrial quality-control strategies. Complementing these pharmacological insights, recent investigations by Sommariva and colleagues have highlighted the interplay between oxidized lipids, inflammation, and mitochondrial dysfunction in cardiovascular pathology. Their findings demonstrate that oxidized LDL–driven signaling promotes oxidative stress, disrupts mitochondrial homeostasis, and triggers inflammatory remodeling, thereby contributing to arrhythmogenic and ischemic complications [10].

These results underscore the rationale for combining lipid-lowering therapy with mitochondria-targeted antioxidants, aiming to synergistically suppress oxidative stress, restore mitochondrial function, and improve vascular outcomes.

### 4.4. Emerging Approaches

Advances in gene therapy offer promising strategies to directly correct mtDNA defects. Approaches such as mitoTALENs, mitochondrial zinc finger nucleases (mtZFNs), and emerging mito-CRISPR/Cas9 systems aim to selectively degrade or edit mutant mtDNA, thereby shifting heteroplasmy toward healthy genomes [170,171].

Although mtDNA mutations traditionally present with neuromuscular and metabolic phenotypes, several variants have been increasingly associated with cardiovascular involvement. Pathogenic mutations in MT-ND1, MT-ND5, MT-CO1 and mt-tRNA genes have been linked to hypertrophic and dilated cardiomyopathy, conduction defects, arrhythmogenic phenotypes and left-ventricular non-compaction [172,173]. Preclinical models such as mt-tRNA mutant mice and PolgA “mutator” mice further demonstrate that heteroplasmic mtDNA defects impair oxidative phosphorylation, promote cardiomyocyte loss and accelerate heart failure development [174,175].

These findings provide mechanistic support for mtDNA-targeted therapeutic approaches in cardiovascular disease. The delivery of therapeutic agents to mitochondria remains technically challenging due to limited membrane permeability and organelle specificity. Recent progress in non-viral nanocarrier systems—including mitochondria-targeted liposomes, DQAsomes, polymeric micelles, and other nanoparticle platforms—has improved mitochondrial targeting, stability and intracellular delivery of therapeutic cargo in preclinical cardiovascular settings [116,176,177,178].

In addition to gene-editing tools, mitochondrial transplantation has emerged as a genome-independent strategy to restore mitochondrial function. Transplantation of viable, respiration-competent mitochondria into ischemic myocardium enhances ATP production, stabilizes membrane potential, reduces oxidative stress and improves post-ischemic functional recovery. These effects have been demonstrated in large-animal and small-animal models, including porcine ischemia–reperfusion, pediatric myocardial ischemia, and inflammatory cardiomyopathy [179,180,181]. Collectively, these gene- and mitochondria-based approaches broaden the therapeutic landscape for targeting mitochondrial dysfunction in cardiovascular disease.

Beyond genetic manipulation, pharmacological stimulation of mitochondrial biogenesis—mediated through PGC-1α–NRF1/2 signaling—has emerged as a viable strategy to restore mitochondrial mass and improve energetic resilience in experimental models of heart failure [115].

At the translational level, mitochondria-targeted peptides such as SS-31 (elamipretide) have progressed to clinical evaluation. In preclinical models of pressure-overload-induced heart failure, SS-31 alleviates mitochondrial dysfunction by enhancing fusion dynamics, partly through Sirt3-mediated mechanisms [182]. In human failing hearts, acute elamipretide administration has been shown to improve ETC efficiency and mitochondrial respiration [183]. However, clinical trials in heart failure and mitochondrial disorders have yielded mixed results, likely due to limitations in bioavailability, inter-individual variability in mitochondrial uptake, and the absence of reliable biomarkers for mitochondrial dysfunction [115].

Despite promising preclinical data, major obstacles remain for clinical translation—including limited mitochondrial delivery, off-target effects in mtDNA editing, and patient heterogeneity in mitochondrial pathologies [184,185]. Overcoming these hurdles is essential to bring mitochondria-targeted therapies from bench to bedside.

**Figure 4:**
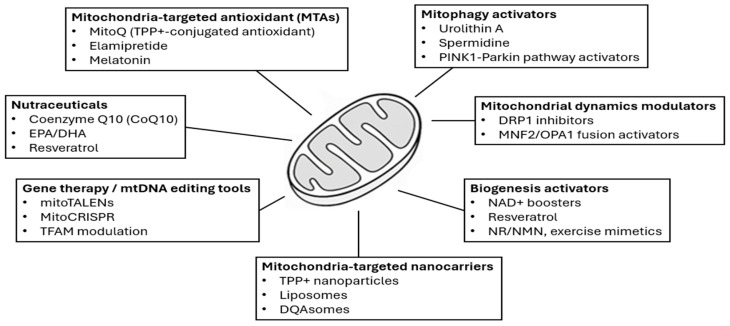
Overview of therapeutic strategies targeting mitochondrial dysfunction in cardiovascular disease.

A comprehensive overview of the therapeutic strategies discussed in this section is summarized in Figure 4.

The figure summarizes eight major therapeutic categories discussed in Section 4, MTAs, mitophagy activators, nutraceuticals, mitochondrial dynamics modulators, biogenesis activators, proteostasis/mtUPR modulators, mitochondria-targeted nanocarriers and gene therapy/mtDNA-editing approaches.

To integrate the mechanistic insights discussed above with their therapeutic and translational implications, a comparative overview of current and emerging strategies targeting mitochondrial dysfunction in cardiovascular disease is summarized in Table 1.

## 5. Current Controversies and Paradoxes in Mitochondrial Cardiovascular Biology

Mitochondria sit at the heart of cardiovascular pathology, yet our field still wrestles with tensions that blur both mechanism and translation. One persistent complication is the patient-specific context: aging, metabolic status, co-morbidities, and co-medications can rewire bioenergetics, redox tone, and Ca^2+^ dynamics, thereby muting signals of cardioprotection that appear robust in young, lean, tightly controlled models. Diabetes is a prime example—by reshaping substrate utilization, depressing oxidative efficiency, and perturbing mitochondrial Ca^2+^ handling, it changes how the heart engages or resists mitochondria-targeted interventions and helps explain why promising preclinical paradigms have struggled in trials [186,187].

Another major paradox concerns mROS. While excessive mROS production is clearly implicated in oxidative damage, energetic failure, and cell death, low-to-moderate levels of mROS act as essential second messengers that activate adaptive transcriptional programs and enhance cellular stress resistance, a phenomenon often referred to as mitohormesis. This dual role complicates therapeutic strategies aimed at broadly suppressing oxidative stress, as indiscriminate antioxidant approaches may inadvertently disrupt beneficial redox signaling and contribute to inconsistent or neutral clinical outcomes [8,188].

Similar ambiguity characterizes the role of mitophagy in cardiac disease. Insufficient mitophagy promotes the accumulation of dysfunctional mitochondria and sustained oxidative stress, whereas excessive or prolonged mitophagy may deplete mitochondrial content below the energetic requirements of cardiomyocytes, thereby impairing contractile function. These opposing effects underscore that mitophagy is not inherently protective or detrimental but must be precisely balanced according to disease stage and metabolic demand, posing a significant challenge for therapeutic modulation [26].

mPTP represents another illustrative paradox. Although mPTP opening is a well-established mediator of ischemia–reperfusion injury and its inhibition is consistently cardioprotective in experimental models, clinical trials using cyclosporine A failed to demonstrate significant benefit. This discrepancy likely reflects the complexity of human ischemic injury, including timing constraints, insufficient myocardial drug exposure, redundancy of injury pathways, and the confounding influence of comorbidities and co-medications, highlighting the limitations of targeting a single mitochondrial node in advanced disease [187,189,190].

Finally, metabolic remodeling in heart failure exemplifies the context-dependent nature of mitochondrial dysfunction. Although a shift from fatty acid oxidation toward increased glucose utilization may initially improve oxygen efficiency, prolonged metabolic inflexibility reduces ATP yield and limits adaptive capacity. Recent evidence indicates substantial heterogeneity across heart failure phenotypes and disease stages, challenging simplified models of uniform metabolic reprogramming and suggesting that metabolic interventions may be beneficial only within specific clinical contexts [112,191].

Collectively, these controversies emphasize that mitochondrial pathways in cardiovascular disease are highly dynamic and context dependent. Rather than representing linear therapeutic targets, mitochondrial processes operate within tightly regulated networks that can exert protective or detrimental effects depending on timing, disease stage, and patient-specific factors. These considerations support the emerging view that effective mitochondrial therapies will require patient stratification and fine-tuning of mitochondrial pathways, rather than uniform activation or inhibition, to achieve meaningful and durable cardioprotection.

## 6. Conclusions and Future Perspectives

This review highlights mitochondrial quality control as a central and unifying mechanism in cardiovascular pathophysiology. Rather than acting as isolated processes, mitochondrial dynamics, mitophagy, biogenesis, and proteostasis are tightly interconnected and collectively determine mitochondrial integrity, signaling capacity, and cellular survival in the stressed heart.

We further emphasize how mitochondrial dysfunction extends beyond bioenergetic failure to actively drive oxidative stress, inflammatory signaling, calcium dysregulation, and maladaptive metabolic remodeling.

Importantly, recent evidence discussed here supports the concept that therapeutic strategies aimed at fine-tuning—rather than simply activating or inhibiting—individual MQC pathways may offer superior cardioprotection. By integrating molecular mechanisms with emerging therapeutic approaches, this work provides a framework for understanding how coordinated mitochondrial quality control governs disease progression and represents a promising target for precision interventions in cardiovascular disease.

## Figures and Tables

**Table 1:** Mitochondria-targeted therapeutic strategies in cardiovascular disease.

Therapy/Class	Primary Mitochondrial Target	Evidence Level	Clinical Status	Key Limitations
Cardiolipin stabilizers (SS-31/Elamipretide)	Stabilization of cristae structure and ETC supercomplexes; reduction in mitochondrial ROS	Strong preclinical (I/R injury, HF models)	Early-phase clinical trials; mixed or modest efficacy	Variable tissue uptake; modest functional improvements; need for larger, adequately powered trials
Mitochondria-targeted antioxidants (MitoQ, MitoTEMPO)	Mitochondrial ROS detoxification; improved redox homeostasis	Robust preclinical	Limited human evidence (small studies; surrogate endpoints)	Dose/duration optimization needed; limited data on hard clinical outcomes
Mitophagy activators (Urolithin A, Spermidine; BNIP3/FUNDC1 pathways)	Enhancement of PINK1/Parkin-dependent and receptor-mediated mitophagy	Strong preclinical	Early human biomarker modulation; no outcome-level data	Translation to clinical endpoints remains limited; long-term effects unknown
Biogenesis activators (AMPK–SIRT–PGC-1α axis)	Promotion of mitochondrial biogenesis and oxidative capacity	Strong preclinical	Early clinical signals	Heterogeneous responses depending on metabolic context and disease stage
SGLT2 inhibitors	Indirect improvement of mitochondrial efficiency, redox state, and fusion–fission balance	Preclinical + strong clinical outcome evidence (RCTs)	Approved therapy for heart failure	Mitochondrial effects are indirect; inter-individual variability in response
MCU–mPTP modulators	Control of Ca^2+^ influx (MCU) and prevention of pathological mPTP opening	Strong mechanistic and preclinical rationale	Clinical translation attempted for mPTP (e.g., cyclosporin A) with largely neutral results	Lack of selective and safe chronic modulators; timing and delivery challenges; pathway redundancy in humans
Inflammation modulators (NLRP3 inhibitors, cGAS–STING blockers)	Inhibition of mtDNA-driven inflammasome activation and innate immune signaling	Strong preclinical	Early translational stage	Safety concerns (potential immune suppression); need for patient stratification and biomarkers
Dynamics modulators (DRP1 inhibitors)	Reduction in excessive mitochondrial fission; restoration of fusion–fission balance	Preclinical	Early development; no approved agents	Specificity and off-target effects; long-term safety unknown

## Data Availability

No new data were created or analyzed in this study. Data sharing is not applicable to this article.
